# Smoking during pregnancy and its effect on placental weight: a Mendelian randomization study

**DOI:** 10.1186/s12884-024-06431-0

**Published:** 2024-04-04

**Authors:** Annika Jaitner, Marc Vaudel, Krasimira Tsaneva-Atanasova, Pål R. Njølstad, Bo Jacobsson, Jack Bowden, Stefan Johansson, Rachel M. Freathy

**Affiliations:** 1 Department of Clinical and Biomedical Sciences, Faculty of Health and Life Sciences, University of Exeter, Exeter, UK; 2 Mohn Center for Diabetes Precision Medicine, Department of Clinical Science, University of Bergen, Bergen, Norway; 3 Department of Genetics and Bioinformatics, Division of Health Data and Digitalization, Norwegian Institute of Public Health, Oslo, Norway; 4 Computational Biology Unit, Department of Informatics, University of Bergen, Bergen, Norway; 5 Department of Mathematics and Statistics, Faculty of Environment, Science and Economy, University of Exeter, Exeter, UK; 6 EPSRC Hub for Quantitative Modelling in Healthcare University of Exeter, Exeter, UK; 7 Children and Youth Clinic, Haukeland University Hospital, Bergen, Norway; 8 Department of Obstetrics and Gynecology, Institute of Clinical Sciences, Sahlgrenska Academy, University of Gothenburg, Gothenburg, Sweden; 9 Department of Obstetrics and Gynecology, Sahlgrenska University Hospital, Gothenburg, Gothenburg, Sweden; 10 Novo Nordisk Genetics Centre of Excellence, Oxford, UK; 11 Department of Medical Genetics, Haukeland University Hospital, Bergen, Norway

**Keywords:** Smoking, Placental weight, Birth weight, Mendelian randomization, ALSPAC, MoBa

## Abstract

**Background:**

The causal relationship between maternal smoking in pregnancy and reduced offspring birth weight is well established and is likely due to impaired placental function. However, observational studies have given conflicting results on the association between smoking and placental weight. We aimed to estimate the causal effect of newly pregnant mothers quitting smoking on their placental weight at the time of delivery.

**Methods:**

We used one-sample Mendelian randomization, drawing data from the Avon Longitudinal Study of Parents and Children (ALSPAC) (*N* = 690 to 804) and the Norwegian Mother, Father and Child Cohort Study (MoBa) (*N* = 4267 to 4606). The sample size depends on the smoking definition used for different analyses. The analysis was performed in pre-pregnancy smokers only, due to the specific role of the single-nucleotide polymorphism (SNP) rs1051730 (*CHRNA5 – CHRNA3 – CHRNB4*) in affecting smoking cessation but not initiation.

**Results:**

Fixed effect meta-analysis showed a 182 g [95%CI: 29,335] higher placental weight for pre-pregnancy smoking mothers who continued smoking at the beginning of pregnancy, compared with those who stopped smoking. Using the number of cigarettes smoked per day in the first trimester as the exposure, the causal effect on placental weight was 11 g [95%CI: 1,21] per cigarette per day. Similarly, smoking at the end of pregnancy was causally associated with higher placental weight. Using the residuals of birth weight regressed on placental weight as the outcome, we showed evidence of lower offspring birth weight relative to the placental weight, both for continuing smoking at the start of pregnancy as well as continuing smoking throughout pregnancy (change in z-score birth weight adjusted for z-score placental weight: -0.8 [95%CI: -1.6,-0.1]).

**Conclusion:**

Our results suggest that continued smoking during pregnancy causes higher placental weights*.*

**Supplementary Information:**

The online version contains supplementary material available at 10.1186/s12884-024-06431-0.

## Background

Maternal smoking during pregnancy is often described as one of the most modifiable risk factors for adverse pregnancy outcomes [1]. Despite a strong public health message, many women continue to smoke in pregnancy. In the UK, the NHS digital service provides statistics indicating that approximately 8.6% of mothers were known smokers at the time of delivery in the first half of 2023 [2]. Mendelian randomization (MR) studies between smoking during pregnancy and offspring birth weight suggest a causal relationship between smoking during pregnancy and lower birth weight [3–6]. However, the underlying mechanisms remain unclear.

A potential mediator for the effect of smoking on fetal growth is the placenta, which provides oxygen and nutrient transport between mother and fetus [7]. The maternal environment is experienced through the placenta [8]. Additionally, studies have shown that maternal smoking is associated with altered histological morphology and structure, which, for example, can lead to a reduction in vascularization [9, 10]. Such abnormalities and the direct effect of nicotine on the placenta can reduce the maternal and fetal exchange, potentially leading to placental insufficiency [11, 12]. Several observational studies have reported a reduction in placental weight in mothers who smoked in pregnancy or continued to smoke compared to non-smoking mothers [7, 13]. Furthermore, a linear decrease in placental weight with the number of cigarettes smoked per day was observed [13]. In contrast to these findings, Mitsuda et al. observed the highest placental weights for women who continued to smoke in pregnancy compared with those who never smoked or who quit smoking before pregnancy [14]. The apparently conflicting results from observational epidemiological studies linking smoking to placental weight may be due to unmeasured confounding and bias, and were conducted in different populations and with different study designs, making them difficult to compare. Hence, additional approaches are necessary to investigate a potential causal relationship.

**Fig. 1:**
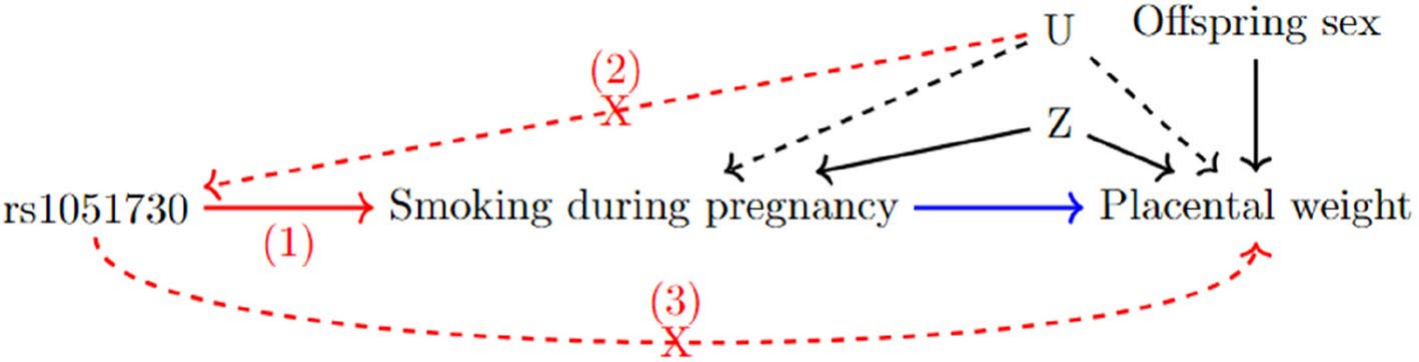
Directed acyclic graph (DAG) to highlight the MR framework. The MR assumptions for the instrumental variable (in this case maternal rs1051730) are shown in red: 1 The instrumental variable needs to be associated with the exposure. 2 The instrumental variable is independent of confounding factors that confound the association of the exposure and the outcome. 3 The instrumental variable is independent of the outcome given the exposure and the confounding factors. The MR analysis estimates the effect between the exposure and the outcome shown in blue. The MR analysis is adjusted for offspring sex and ancestry principal components (and genetic batch variables in MoBa). These are summarised in the measured confounder variable Z. U stands for unmeasured confounders, which we are unable to include in the analysis

One method enabling the inference of causal effects in the presence of confounding is one-sample MR. It is a method, which utilises the natural randomization of inheritance of germline genetic variation from parents to their offspring at conception [15]. We used a genetic variant, single-nucleotide polymorphism (SNP) rs1051730, as the instrumental variable to genetically proxy maternal smoking. Previous studies have shown that each additional copy of the risk allele rs1051730 is associated with higher odds of continuing smoking during pregnancy as well as an increase of about one cigarette per day [16–18]. The SNP is located within the nicotine acetylcholine receptor gene cluster *CHRNA5 – CHRNA3 – CHRNB4* on chromosome 15. The biological relationship to smoking and nicotine dependence supports the association between the SNP and smoking. However, it is important to note that rs1051730 is not associated with smoking initiation [16, 18]. Due to the specific association of rs1051730 with smoking behaviour, we only used data from mothers who smoked before pregnancy to capture continuing smoking compared to stopping smoking in pregnancy. To investigate any causal relationship between maternal smoking during pregnancy and placental weight we used two cohorts: the Avon Longitudinal Study of Parents and Children (ALSPAC) [19, 20] and the Norwegian Mother, Father and Child Cohort Study (MoBa) [21, 22]. Figure 1 shows the directed acyclic graph describing the causal assumptions for our study analysis. Our aim was to improve the understanding of the effect of continuing smoking in pregnancy by investigating the causal relationship between maternal smoking and placental weight.

## Methods

### Study populations

**Fig. 2:**
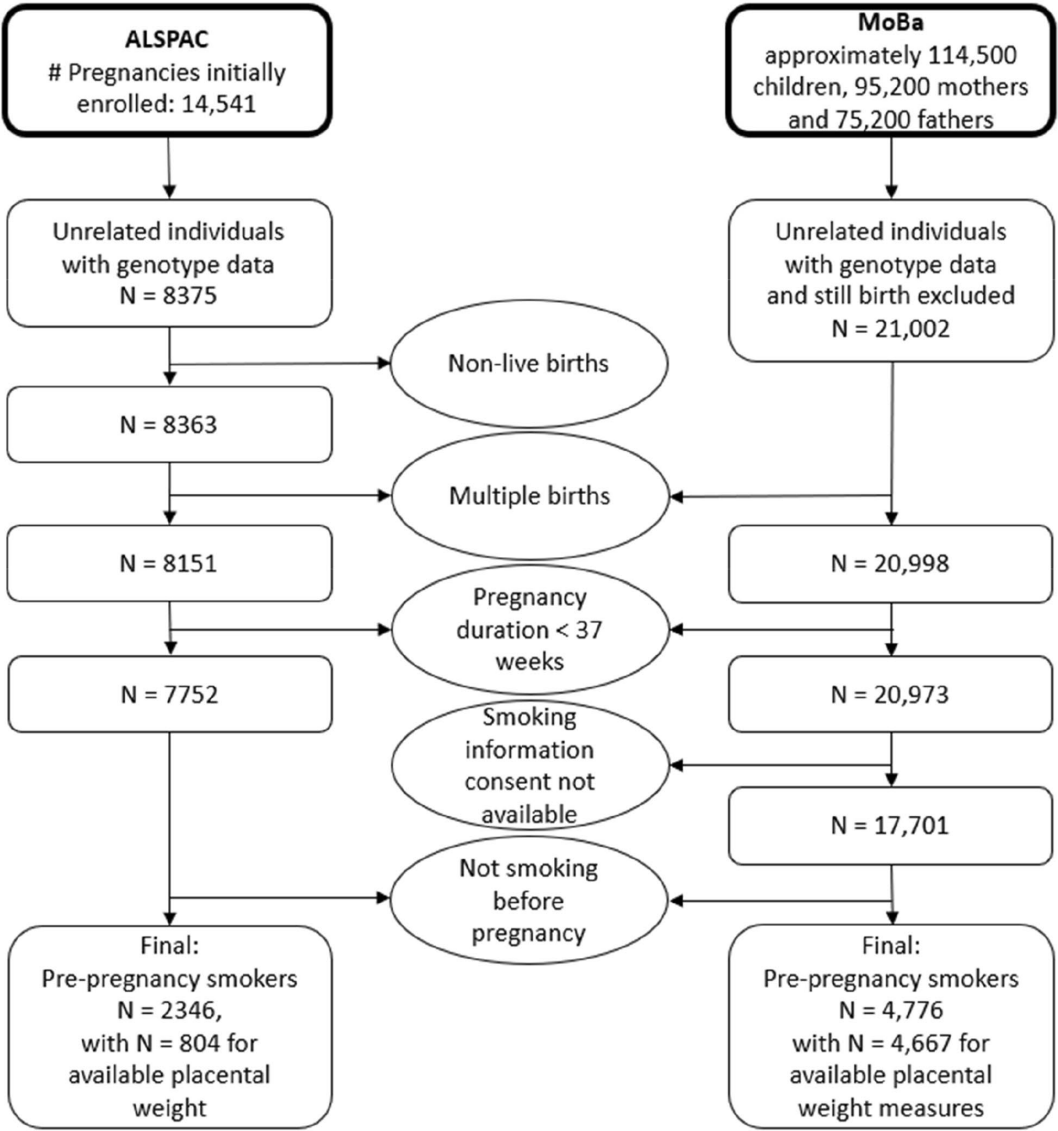
Flowchart to display the exclusion criteria of both the ALSPAC and the MoBa study including sample sizes

We performed our analysis in two different study populations. **ALSPAC** is a prospective longitudinal cohort study [19, 20]. More information on the cohort is given in the supplementary material. Our analysis was performed in unrelated mothers with genetic information for rs1051730 available. Placental weight measures were available for 37% of the records. We excluded multiple births and preterm births (pregnancy duration < 37 weeks). Full details including sample sizes are shown in Fig. 2. After all exclusions, the analysis in pre-pregnancy smokers with available placental weight measures as an outcome was therefore performed in up to 804 individuals in ALSPAC. **MoBa** is a population-based pregnancy cohort study conducted by the Norwegian Institute of Public Health [21, 22]. The study is linked with the **Medical Birth Registry of Norway (MBRN)**, a national health registry containing information about all births in Norway. More detailed information on the cohort and the version used is given in the supplementary material. We restricted the MoBa data to unrelated individuals with genetic information for the mother available. Additionally, we excluded multiple births and preterm births (pregnancy duration < 37 *7 days). Full details including sample sizes are shown in Fig. 2. After all exclusions, there were 4667 pre-pregnancy smokers with available placental weight measures in MoBa, and up to 4606 of these individuals had smoking information relevant for our analyses.

#### Genetic instrument

We instrumented the smoking behaviour using rs1051730, which has shown to be associated with smoking quantity and the inability to quit smoking but not smoking initiation [16–18]. We used the genotype dosage of the genetic variant, rs1051730 as a continuous variable, which for each individual was a number close to 0, 1, or 2, reflecting the number of smoking risk alleles, combined with the probability of having 0, 1, or 2 risk alleles from the genotype imputation. More information on genotyping in both cohorts is described in detail in previously published articles [22, 23].

#### Outcome variable

The main outcome of interest is placental weight measured in grams. In ALSPAC, placental weight measures were obtained directly from obstetric records by research midwives who went back to the handwritten medical records of most patients and abstracted data including all weight measures. In MoBa, data related to pregnancy and birth were standardised and stem from the MBRN. Reporting placenta weight to the MBRN is mandatory and is carried out by the midwife attending the birth. All midwives share curriculum and training regarding the reporting of data, including examination of the afterbirth to the MBRN. The placenta is examined and characteristics of the placenta and umbilical cord, including measurements of the placental weight (untrimmed with the cord and membranes attached) are reported. The method has been unchanged since the inception of the MBRN in 1967. The reporting of these data to the MBRN has been validated, with good inter- and intra-observer agreement, making the data suitable for large scale epidemiological research [24].

#### Exposure variable

The exposures of interest were (i) continuing smoking during pregnancy vs. quitting and (ii) number of cigarettes smoked per day during pregnancy. We used different measures of self-reported smoking variables. Study specific differences are outlined below.

#### Smoking variables of interest in ALSPAC

In ALSPAC, mothers were asked if they smoked before pregnancy. No specific time frame was given in the questionnaire to the mothers. We included everyone in the study who said they consumed tobacco before pregnancy even if this consumption was through other sources than cigarettes, such as pipes and cigars. The frequency of tobacco consumption via cigarettes was by far the highest (97.8% of the mothers who smoked pre-pregnancy said they smoked cigarettes). The following smoking variables were used as exposures in the analysis performed in ALSPAC:

##### Smoking in the first three months of pregnancy

At 18 weeks of gestation the mother was asked whether she smoked in the first three months of pregnancy. This variable is self-reported and retrospective.

##### Smoking in the last two weeks of pregnancy

This information was gained from a questionnaire sent out 8 weeks after the child was born. As for the previous variables smoking refers to any type of tobacco consumption.

##### Number of cigarettes smoked per day

Besides classifying whether a mother smoked or not as a binary variable, the participants were also asked about the number of cigarettes smoked per day in the first three months and the last two weeks of pregnancy. The following categories were given: 0 cigarettes, 1–4, 5–9, 10–14, 15–19, 20–24, 25–29, 30 or more cigarettes. For the analysis the categories were coded with the number of the lower bound of each category.

#### Smoking variables of interest in MoBa

In MoBa, the mothers were asked whether they smoked during the last three months before becoming pregnant. The information for all smoking variables is taken from the MBRN [25]. The following smoking variables were used as exposures in the analysis performed in MoBa:

##### Mother smoking at the beginning of pregnancy

The antenatal health card containing this information is filled out at the first antenatal visit between 6 and 12 weeks of gestation.

##### Mother smoking at the end of pregnancy

The end of pregnancy corresponds to the last trimester (approximately 36 weeks of gestation).

##### Number of cigarettes smoked per day

 This information was recorded for the beginning and the end of pregnancy. In contrast to ALSPAC, the number of cigarettes in MoBa is given in integer values, instead of being grouped into categories. Mothers who stopped smoking in pregnancy and therefore reported that they were not smoking at the beginning and/or the end of pregnancy were coded with 0 cigarettes.

### Mendelian randomization

We performed one-sample MR using individual level data. MR requires three assumptions to hold for rs1051730 to be a valid instrumental variable [15]. The assumptions are graphically highlighted in Fig. 1. Due to the genetic variants being defined at conception we assumed that it is independent of factors confounding the association between smoking during pregnancy and placental weight. We cannot formally test that the genetic instrument is only associated with the outcome through the exposure. However, based on the position of rs1051730 in the genome and therefore likely biological role, we assumed that the third assumption holds as well. We test the association between the SNP and placental weight in mothers who have never smoked to further support that there are no pleiotropic pathways. Additionally, we studied the association between the SNP and various variables in the MoBa study.

For all analyses, we aimed to estimate the causal effect of smoking on placental weight (PW) in mothers who smoked pre-pregnancy (${S}_{pre}$=1). For continuous smoking definitions, our causal estimand was the population average effect of intervening to lower individuals observed smoking level $s$ by 1 cigarette per day.

$${\mathrm{Continuous}\;\mathrm{smoking}}:E\left[{PW(S=s)\vert S}_{pre}=1\right]-E\lbrack PW(S=s-1)\vert S_{pre}=1\rbrack$$

For binary smoking definitions, our causal estimand reflects the population average effect if all mothers continued to smoke versus if all mothers subsequently quit.

$${\mathrm{Binary}\;\mathrm{smoking}}:E\left[{PW(S=1)\vert S}_{pre}=1\right]\;-\;E\left[{PW(S=0)\vert S}_{pre}=1\right]$$

In each case, we impose a fourth identifying assumption of homogeneity, meaning that the causal effect does not vary across levels of a single instrument, nor across instruments. For all analysis a two-stage regression approach was used. In the continuous smoking exposure case, the smoking variable (S) was firstly regressed on rs1051730 (G) and adjusted for known confounders or competing exposures (Z) via a linear model:

1 $$S\;\vert{(S}_{pre}=1),G,Z=\alpha_0+\alpha_1G+\alpha_2Z+\epsilon_{S,}$$

to furnish a genetically predicted smoking variable ($\widehat{S})$. Note that this first stage regression does not require any placental weight measurements. We therefore performed the regression in all pre-pregnancy smoking mothers with available smoking information during pregnancy. The sample sizes used for this stage are given in Table 2. Secondly, PW was regressed on $\widehat{S}$:

2 $$PW|{(S}_{pre}=1),\widehat{S},Z={\beta }_{0}+{\beta }_{1}\widehat{S}+{ \beta }_{2}Z+{\epsilon }_{PW}$$

We adjusted all our analyses for offspring sex and ancestry principal components, which is reflected by Z in the equations above. We performed sensitivity analyses adjusting for additional potential confounders (Supplementary SFigure 2 and SFigure 3). For binary smoking exposure variables, we performed a logistic regression in the first stage. The estimation of the standard error of the causal estimate (${\beta }_{1}$) accounts for first stage uncertainties.

#### Residuals of z-score birth weight on z-score placental weight

We performed a final analysis by incorporating both birth and placental weight into a single outcome variable, thereby taking into account their relationship. Using the residuals from the regression of birth weight on placental weight can be used as a measure of placental efficiency [26].

1. We firstly generated z-scores using generalised additive models for location, scale and shape from the gamlss R-package [27, 28]:
  - of placental weight adjusting for gestational duration in female offspring ($P{W}_{Zf}$);
  - of placental weight adjusting for gestational duration in male offspring ($P{W}_{Zm}$);
  - of birth weight adjusting for gestational duration in female offspring ($B{W}_{Zf}$ );
  - of birth weight adjusting for gestational duration in male offspring ($B{W}_{Zm}$).

This resulted in adjusted z-scores of birth weight $B{W}_{Z}=(B{W}_{Zm}, B{W}_{Zf})$ and placental weight $P{W}_{Z}=(P{W}_{Zm}, P{W}_{Zf})$. The scores were derived from the individual level data within the ALSPAC and the MoBa study separately.

- 2) We then regressed $B{W}_{Z}$ on $P{W}_{Z}$:
- 3 $$B{W}_{Z}={\gamma }_{0}+{\gamma }_{1}P{W}_{Z}+{\epsilon }_{B{W}_{Z}.}$$
- 3) Next, we took the estimated residuals ($\widehat{R}$) from the equation in step 2: $\widehat{R}=B{W}_{Z}-\widehat{B{W}_{Z}.}$
- 4) Finally, we used the residuals from step 3 as the outcome in an MR analysis with a binary smoking exposure S, applying the two stage approach below:
- 4 $$logit(Pr(S=1|({S}_{Pre}=1),G,Z))={\alpha }_{0}+{\alpha }_{1}G+{\alpha }_{2}Z+{\epsilon }_{S}$$
- 5 $$\widehat{R}|{(S}_{Pre}=1),\widehat{S},Z={\beta }_{0}+{\beta }_{1}Z+{\epsilon }_{\widehat{R}}.$$

This enabled us to estimate the causal effect of maternal smoking on birth weight relative to placental weight.

#### Adjustment variables and meta-analysis 

We adjusted all analysis for offspring sex and principal components to account for population stratification (first 5 in ALSPAC and first 10 in MoBa). All analysis in MoBa were additionally adjusted for genetic batch variables. After performing the MR study in ALSPAC and in MoBa, we meta-analysed the results from smoking at the beginning of pregnancy and smoking at the end of pregnancy. The Q statistics (on 1df) (STable 1) provided no evidence to refute the null hypothesis that causal estimates derived from ALSPAC and MoBa pertained to different underlying quantities. We therefore combined them using an inverse variance weighted fixed effect model to produce an overall estimate.

## Results

### Study population characteristics

Table 1 shows clinical characteristics in the datasets used for the analysis from both the ALSPAC and the MoBa study.

**Table 1:** Descriptive statistics of the ALSPAC and the MoBa cohort samples used for the analysis. For the continuous variables mean and standard derivation are displayed. For the categorical variables a percentage is given. If there are two variable descriptions given in one row, then the first one corresponds to the ALSPAC study and the second one to the MoBa study

Variable	Mean (SD) / Percentage		*N* without missing	
**Study**	**ALSPAC**	**MoBa**	**ALSPAC**	**MoBa**
Birth weight (g)	3402 (486)	3674 (484)	2346	4770
Placental weight (g)	654 (131)	689 (145)	804	4667
Gestational duration (weeks in ALSPAC/days in MoBa)	39.8 (1.3)	282 (9)	2346	4776
Mothers age (years)	26.8 (5)	28.7 (4.7)	2346	4776
Mothers height (cm)	164 (7)	168.3 (5.8)	2102	4735
Mothers pre-pregnancy weight/ at the beginning of pregnancy (kg)	62 (11)	69 (13)	2011	4660
Offspring (male %)	50.8	50.3	2346	4776
Smoking in the first three month of pregnancy/ Smoking at the beginning of pregnancy (yes %)	72.4	35	2346	4715
Smoking in the last two weeks of pregnancy/Smoking at the end of pregnancy (yes %)	57.9	17.5	2032	4580
Year of delivery	1991–1993	1999–2009	2346	4776

### SNP-exposure association in ALSPAC and MoBa

The results for the association between the different smoking exposures and the genetic instrument rs1051730 are shown in Table 2. This corresponds to the first stage of the MR. For all the different smoking variables the SNP is a strong instrument showing that each additional risk allele increases the likelihood of continuing smoking in pregnancy as well as the quantity.

**Table 2:** Effect estimates of the smoking variables regressed on the genetic instrument as shown in Eq. (1). The analysis is adjusted for offspring sex and the principal components (and genetic batch variables in MoBa). For the first 4 rows a logistic regression is used due to the smoking variables being binary. The effect estimate in the first column gives the odds of continuing smoking in pregnancy per additional risk allele of rs1051730. For the last 4 rows a linear regression is used. The effect estimate in the first column gives the change in the number of cigarettes smoked per day per each additional risk allele of rs1051730

Smoking variable	Effect Estimate	Lower 95% CI	Higher 95% CI	*P*-value	F-statistic	*N*	Study
Smoking in the first three months of pregnancy	1.27	1.11	1.46	0.0009	11.1	2346	ALSPAC
Smoking in the last two weeks of pregnancy	1.25	1.09	1.43	0.0015	10	2032	ALSPAC
Smoking at the beginning of pregnancy	1.15	1.04	1.27	0.0016	9.9	4711	MoBa
Smoking at the end of pregnancy	1.27	1.13	1.43	0.00004	17.1	4576	MoBa
# of cigarettes smoked per day in the first three month	0.88	0.23	1.338	0.0001	14.7	2312	ALSPAC
# of cigarettes smoked per day in the last two weeks	0.87	0.25	1.36	0.0004	12.7	2025	ALSPAC
# of cigarettes smoked per day at the beginning	0.45	0.11	0.67	0.00003	17.2	4360	MoBa
# of cigarettes smoked per day at the end	0.25	0.06	0.37	0.00006	16	4440	MoBa

### Binary smoking exposure

**Fig. 3:**
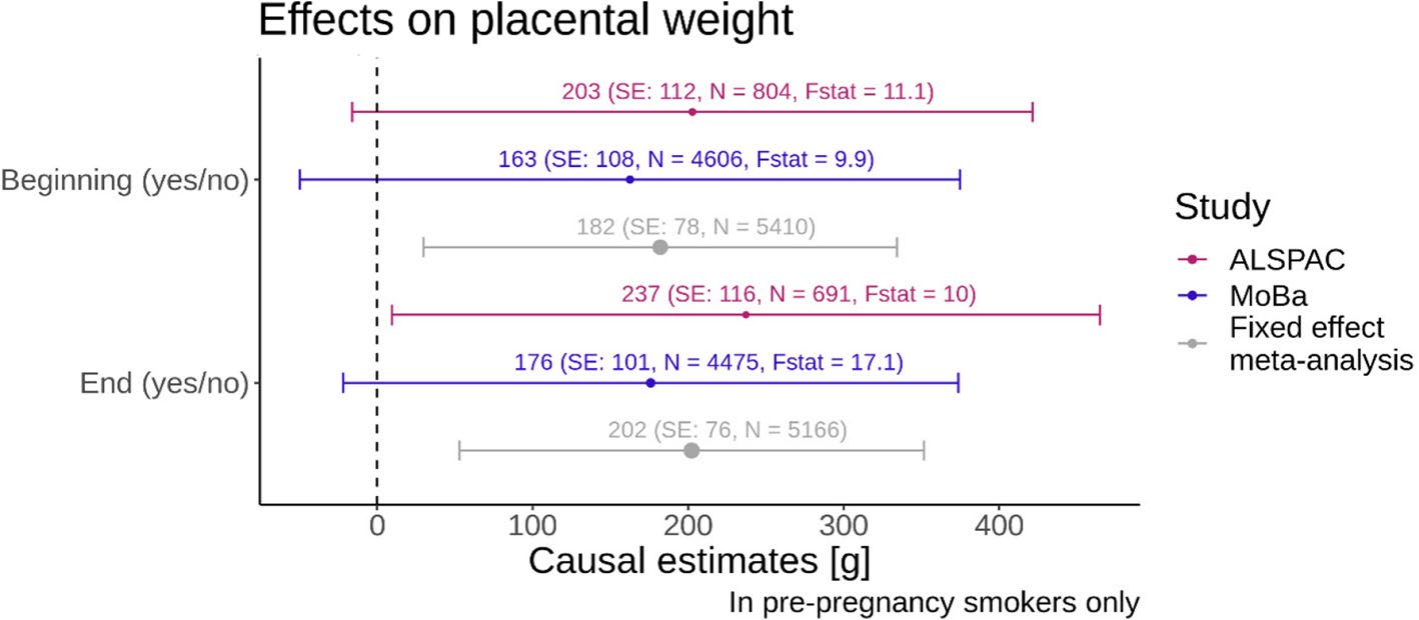
Forest plot with binary smoking variables on the y-axis and the causal estimate from the MR with placental weight as the outcome on the x-axis. The colours indicate the results for the different studies and the fixed effect meta-analysis. The bars indicate the 95% confidence intervals. The F-statistics from the first stage of the MR analysis are displayed alongside with the sample size N for each analysis. The size of the dot of the point estimate for each analysis is proportional to 1/SE

In the fixed effect meta-analysis, we observed that mothers who continued smoking in pregnancy had, on average, a 182 g (95% CI: [29,335]) higher placental weight compared with those who stopped smoking at the beginning of pregnancy. The F-statistic as a measure of the strength of the instrument was 11.1 in ALSPAC and 9.9 in MoBa for the analysis at the beginning of pregnancy, which is very close to the minimum F-Statistic of 10 suggested in the literature [15, 29]. In MoBa, the F-Statistic, 17.1, was higher for the analysis with the smoking at the end of pregnancy exposure. In ALSPAC, the F-Statistic did not change much for the different time points of smoking in pregnancy as the exposure. For both ALSPAC and MoBa, and the meta-analysis similar effect sizes were evident for the analysis at the end of pregnancy (meta-analysis: 202 g, 95% CI: [53,351]) compared to the analysis with the smoking exposure being measured at the beginning of pregnancy. Results for the MR study in pre-pregnancy smokers in ALSPAC and in MoBa as well as the fixed effect meta-analysis for a binary smoking exposure are displayed in Fig. 3.

### Cigarettes smoked per day exposure

**Fig. 4:**
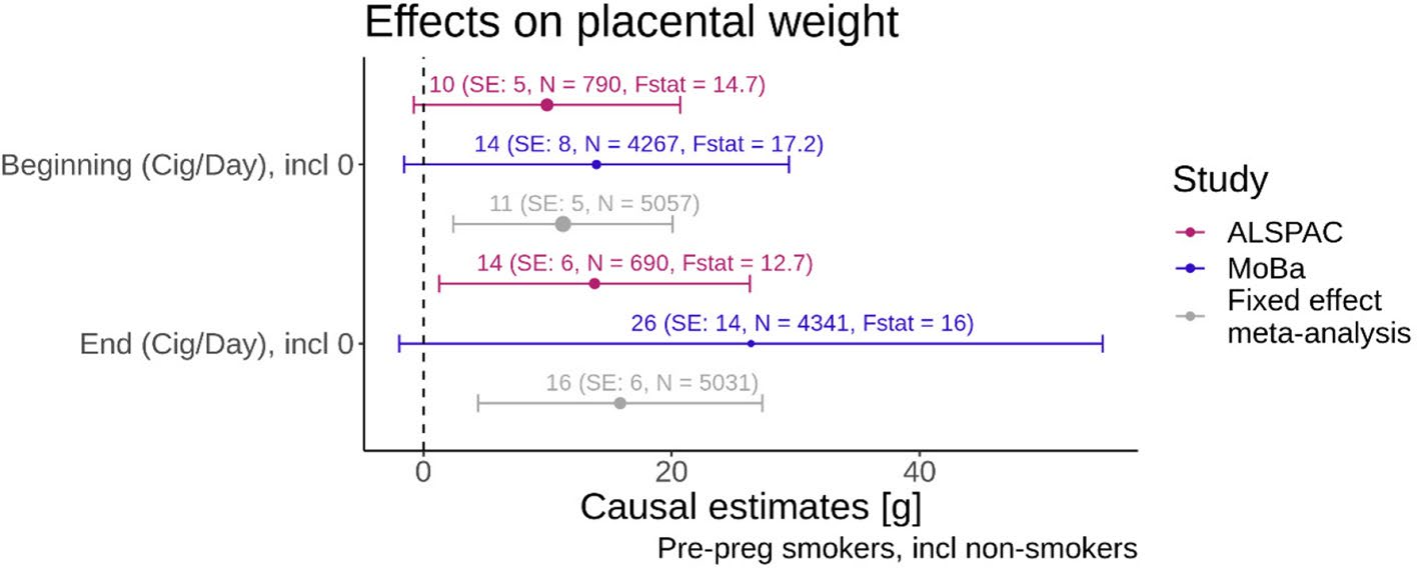
Forest plot with smoking quantity variables on the y-axis and the causal estimate from the MR with placental weight as the outcome on the x-axis. The colours indicate the results for the different studies and the fixed effect meta-analysis. The bars indicate the 95% confidence intervals. The F-statistics from the first stage of the MR analysis are displayed alongside with the sample size N for each analysis. The size of the dot of the point estimate for each analysis is proportional to 1/SE

The meta-analysis results indicated an increase of 11 g (95% CI: [1,21]) in placental weight for each additional cigarette smoked at the beginning of pregnancy amongst the mothers who smoked before pregnancy. Each additional cigarette at the end of pregnancy caused an increase in placental weight of 16 g (95% CI: [4,28]). Figure 4 shows the results of this MR study. The effects in ALSPAC and MoBa were consistent with the meta-analysis. For these analyses the F-statistics were slightly higher than for the binary analysis.

### Residual z score analysis

**Fig. 5:**
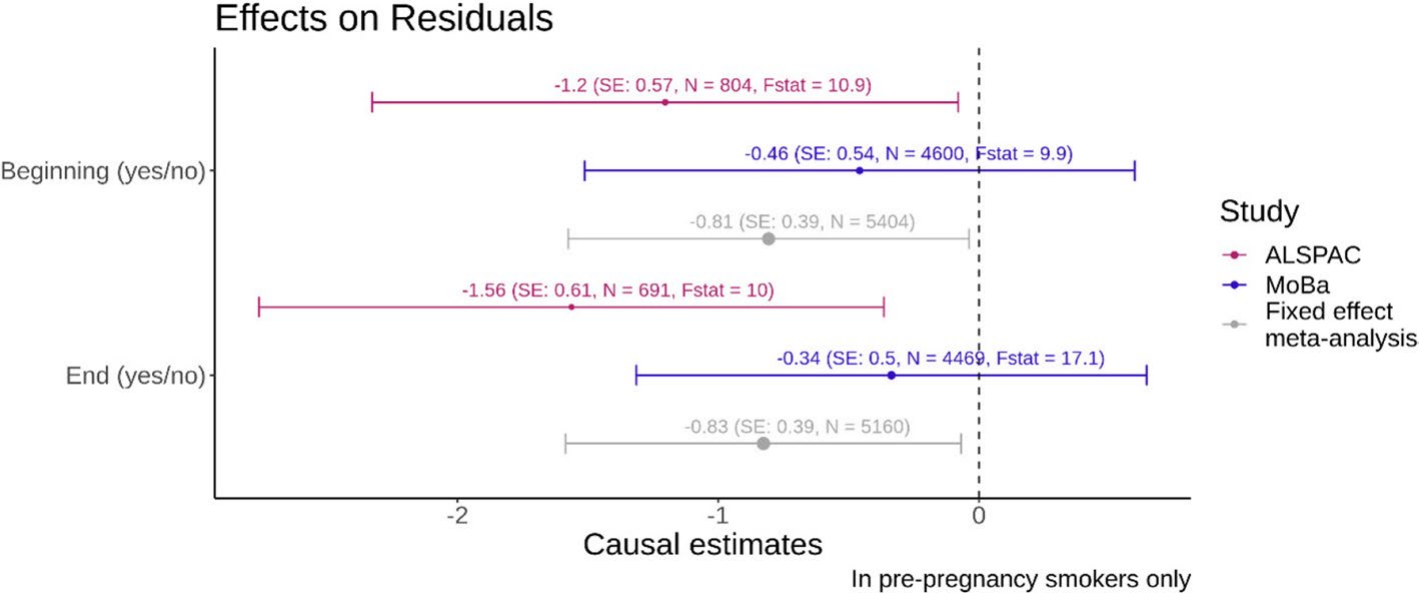
Forest plot with binary smoking variables on the y-axis and the causal estimate from the MR with the residuals of the regression of adjusted z-score birth weight on adjusted z-score on placental weight as the outcome on the x-axis. The effect sizes reflect the change in z-score birth weight adjusted for z-score placental weight for continuing smoking vs quitting in pregnancy. The colours indicate the results for the different studies and the fixed effect meta-analysis. The bars indicate the 95% confidence intervals. The F-statistics from the first stage of the MR analysis are displayed alongside with the sample size N for each analysis. The size of the dot of the point estimate for each analysis is proportional to 1/SE

For both ALSPAC and MoBa, negative point estimates were obtained for the MR with the residuals of the regression of adjusted birth weight on adjusted placental weight as outcome (see Fig. 5). This indicated that for mothers who continue to smoke, their offspring birth weights tend to be lower relative to the placental weight. Christians et al. [26] suggest using the residuals from a regression of birth weight on placental weight as a measure of placenta efficiency. In this context, our results suggest that continuing smoking during pregnancy is causally associated with a lower placenta efficiency.

### Sensitivity analyses

We performed sensitivity analyses to check the assumptions of the MR analysis. In the ALSPAC data, there was no evidence of association between the SNP and placental weight in mothers who have never smoked. The linear regression of placental weight on rs1051730 in never smokers (*N* = 1465) yielded an effect estimate of 3 g per smoking risk allele (95% CI: [-7,13]). We also saw no association between the SNP and various variables that could affect placental weight and therefore are a potential for pleiotropic pathways (supplementary SFigure 1).

All our analysis presented in this paper were adjusted for offspring sex and ancestry principal components. We believe offspring sex is a competing exposure and therefore the adjustment will increase the precision of our analysis. Nevertheless, we performed sensitivity analyses adjusting for different sets of potential confounders and showed consistently an increased placental weight for mothers who continue to smoke in pregnancy (Supplementary SFigure 2 and SFigure 3).

## Discussion

Using an MR approach, we have provided evidence that continuing smoking during pregnancy causes higher placental weights for term born babies. The results were consistent for both the binary exposure of continuing vs quitting smoking and number of cigarettes smoked per day in two independent cohorts.

Given the well-established relationship between maternal smoking in pregnancy and lower birth weight, it is plausible that smoking would lead to lower placental weights due to an impairment of the placental function. Zdravkovic et al. [30] stated the likelihood of tobacco exposure reducing the blood flow between mother and child thereby causing a hypoxic environment for the fetus and this could be manifested in decreased placental and fetal growth as oxygen binding is essential for the development of these organs. However, our findings are more in line with a compensatory effect. The placenta might grow larger relative to birth weight to meet the oxygen demands of the fetus and to restore oxygen binding sites. This hypothesis is supported by our findings of the residual analysis, which showed lower birth weights relative to the placental weight for mothers who continue to smoke vs those who quit smoking in pregnancy. The impact of a hypoxic environment on the placenta has been studied in animal models with conflicting results [31]. For example, increased placental weights with a reduced fetal weight were seen in guinea pigs when exposed to chronic mid gestation 10.5% hypoxia [32] and observed in mice for a chronic early 13% hypoxia [33]. Studies in rats have reported that under chronic 13–14% hypoxia in early gestation, an increased placental weight was detected, but without any change in fetal weight [34, 35]. This suggests that in some conditions the placenta might be able to adapt and compensate for the hypoxic environment, but in other situations, the enhanced placental growth (and therefore placental weight increase) limits other factors of the fetal development process. Placental weight is often used to proxy placental function [36], but as discussed above this is not a straightforward relationship and needs to be carefully interpreted. Additionally, placental weight is a combination of several components including size, surface area and thickness. Both abnormally higher and lower placental weights are associated with increased risk of pregnancy complications [31]. Further explorations of the placenta, placenta functioning and efficacy and how to quantify these are necessary. However, sample sizes of such studies are currently limited and a MR study to investigate causality was not feasible.

Due to the properties of the MR method, adjusting for covariates is not strictly necessary but can increase precision. However, it is important to only adjust for variables that, one is confident about, act as a confounder to the exposure and the outcome variable or a competing exposure. Therefore, we adjusted all our analysis for offspring sex and the population stratification via principal components. There are various other covariates that we could have adjusted for, like, for example, gestational duration. However, it is possible that gestational duration acts as a mediator for smoking in pregnancy and placental weight rather than a confounder. Adjusting for gestational duration could then induce collider bias. In the supplementary material (SFigure 3) we showed in a sensitivity analysis that additionally adjusting the MR analysis for different sets of covariates, which are potential confounders of the relationship between the smoking exposure and placental weight, were consistent with the results in the main paper.

One of the limitations of our study is that the available sample size of mothers who smoke before pregnancy was limited. Hence, this leads to large uncertainties surrounding the magnitude of the effect on placental weight. However, this study comprises two of the biggest mother child birth cohorts available. A second limitation was the inevitable differences in variable measurements between the cohorts. For example, information about the number of cigarettes smoked per day was recorded differently in ALSPAC (categories) and in MoBa (integer values). However, in MoBa, mothers tended to report 5 or 10 cigarettes per day instead of integer values in between, which reduces the differences in this variable between the two cohorts. Additionally, instrumental variable analyses are robust to measurement errors as the expected number of cigarettes smoked is based on the genetic information used for the analysis. There was little heterogeneity between the cohorts, and there was good agreement across various analysis models, which strengthens our results despite measurement differences between the cohorts. Another limitation is that all smoking information from the mothers was self-reported data. The strong public health message on smoking might potentially lead to underreporting of smoking in pregnancy. However, a validation of self-reported smoking was performed in a subset of the MoBa participants and revealed that daily smoking prevalence increased only slightly, from 9 to 11%, when investigating cotinine concentrations, suggesting that self-reported smoking is a valid marker for tobacco exposure in MoBa [37]. It is also important to recognize that the smoking variables at the end of pregnancy only capture whether the mother smoked at this timepoint but for non-smokers it does not give insight into when in pregnancy the mother stopped smoking.

One of the strengths of our study is the use of rs1051730 which has very robust statistical evidence for association with smoking cessation and smoking quantity. There is also strong biological evidence for this association as rs1051730 is in the nicotine acetylcholine receptor gene cluster *CHRNA5-CHRNA3-CHRNB4.* Rare variant burden associations have implicated all three of these genes as important in influencing smoking quantity [38].

## Conclusions

In conclusion, we have provided evidence to support a causal effect of continued maternal smoking in pregnancy on increased placental weight. Using the MR approach, our study adds to existing evidence on the relationship between placental weight and maternal smoking, which until now included inconsistent results from observational studies that are more susceptible to bias and confounding by unmeasured variables. Our work supports a mechanism whereby maternal smoking leads to a compensatory increase in placenta weight, but further investigations on maternal smoking, birth weight and placental properties are necessary to better understand mediation effects or other forms of interactions between these three components.

### Supplementary Information

**Supplementary Material 1.**

## Data Availability

The data in ALSPAC is fully available, via managed systems, to any researchers. The managed system is a requirement of the study funders, but access is not restricted on the basis of overlap with other applications to use the data or on the basis of peer review of the proposed science. The ALSPAC data management plan describes in detail the policy regarding data sharing, which is through a system of managed open access. The following steps highlight how to apply for access to the data included in this paper and all other ALSPAC data. (1) Please read the ALSPAC access policy, which describes the process of accessing the data and samples in detail and outlines the costs associated with doing so. (2) You may also find it useful to browse the fully searchable ALSPAC research proposals database, which lists all research projects that have been approved since April 2011. (3) Please submit your research proposal for consideration by the ALSPAC Executive Committee. You will receive a response within 10 working days to advise you whether your proposal has been approved. If you have any questions about accessing data, please email alspac-data@bristol.ac.uk. Data from the Norwegian Mother, Father and Child Cohort Study and the Medical Birth Registry of Norway used in this study are managed by the national health register holders in Norway (Norwegian Institute of public health) and can be made available to researchers, provided approval from the Regional Committees for Medical and Health Research Ethics (REC), compliance with the EU General Data Protection Regulation (GDPR) and approval from the data owners. The consent given by the participants does not open for storage of data on an individual level in repositories or journals. Researchers who want access to data sets for replication should apply through helsedata.no. Access to data sets requires approval from The Regional Committee for Medical and Health Research Ethics in Norway and an agreement with MoBa.

## Abbreviations

ALSPAC: Avon Longitudinal Study of Parents and Children
BW: Birth weight
CI: Confidence interval
MBRN: Medical Birth Registry of Norway
MoBa: Norwegian Mother, Father and Child Cohort Study
MR: Mendelian randomization
PW: Placental weight
SD: Standard deviation
SNP: Single nucleotide polymorphism
